# The Utility of Advanced Cardiovascular Imaging in Cancer Patients—When, Why, How, and the Latest Developments

**DOI:** 10.3389/fcvm.2021.728215

**Published:** 2021-09-03

**Authors:** Christopher Yu, Faraz Pathan, Timothy C. Tan, Kazuaki Negishi

**Affiliations:** ^1^Nepean Clinical School, University of Sydney, University of Sydney, Sydney, NSW, Australia; ^2^Cardiology Department, Nepean Hospital, Sydney, NSW, Australia; ^3^Cardiology Department, Blacktown Hospital, Sydney, NSW, Australia

**Keywords:** cardio-oncology, cardiotoxicity, cardiac imaging, echocardiography, cardiac magnetic resonance imaging, global longitudinal strain, cardiac computer tomographic imaging, cancer therapeutics related cardiac dysfunction

## Abstract

Cardio-oncology encompasses the risk stratification, prognostication, identification and management of cancer therapeutics related cardiac dysfunction (CTRCD). Cardiovascular imaging (CVI) plays a significant role in each of these scenarios and has broadened from predominantly quantifying left ventricular function (specifically ejection fraction) to the identification of earlier bio-signatures of CTRCD. Recent data also demonstrate the impact of chemotherapy on the right ventricle, left atrium and pericardium and highlight a possible role for CVI in the identification of CTRCD through tissue characterization and assessment of these cardiac chambers. This review aims to provide a contemporary perspective on the role of multi-modal advanced cardiac imaging in cardio-oncology.

## Introduction

Heart disease and cancer are the two major causes of morbidity and mortality, accounting for over 70% of medically related deaths globally (1). The mortality risk due to cardiovascular complications is nearly four times higher in cancer patients compared to the general population (2). It is highest within the first year after cancer diagnosis and remains persistently elevated in cancer survivors even after the completion of treatment (2). The lifetime risk of cancer therapeutics related cardiac dysfunction (CTRCD) from cancer treatment can be increased up to 15-fold (3). Furthermore, the presence of cancer is independently associated with structural, functional, and tissue characteristic changes (4). Hence, cardiac risk stratification, identification of CTRCD due to cancer therapy and predicting cardiac recovery are important management goals in cancer patients. We define CTRCD for the purposes of this review as the direct effect of cancer treatment on the heart structure, function, and acceleration of coronary artery disease.

Cardiac imaging plays an important role in the diagnosis, management, and prognostication in patients with CTRCD (Supplementary Table 1). LVEF has been the most validated and commonly utilized parameter for the assessment of LV systolic function in CTRCD. However, the traditional approach of using left ventricular ejection fraction (LVEF) as a measure of CTRCD is now believed to be inadequate as changes in LVEF are a late manifestation of CTRCD. A desire to intervene earlier has led to a renewed focus on identifying early biosignatures of CTRCD which predate changes LVEF. This review aims to provide a contemporary perspective on the role of multi-modal cardiac imaging in the diagnosis and management of CTRCD.

## Echocardiography

### Baseline Cardiovascular Risk Stratification, Identification of CTRCD and Predicting Recovery

Echocardiography is the primary imaging modality of choice for baseline cardiac function assessment by American and European society consensus statements (5–9). The latest guidelines by the European Society of Medical Oncology (ESMO) have given a IA recommendation that all patients undergoing anticancer therapy associated with LV dysfunction should have a baseline LVEF assessment (5). Patients with impaired LVEF at baseline are at highest risk of cardiotoxicity from anticancer therapy.

While echocardiography is a good first line investigation due to its accessibility, low cost, and lack of any radiation, feasibility of high-quality echocardiographic imaging may also be limited by patient body habitus, radiation therapy, or recent surgery (e.g., mastectomy). In these situations, cardiac magnetic resonance imaging (CMR) or contrast echocardiography can overcome some of these imaging limitations and provide a more accurate assessment of LVEF (10).

#### Left Ventricle

Widely utilized standard 2D echocardiographic methods of LVEF assessment are based on geometrical assumptions which limits accuracy and reproducibility. Three-dimensional (3D) echocardiography overcomes these limitations and is currently recommended for the assessment of LV systolic function (7, 11). Recent developments which allow for semi-automated assessment of 3D LVEF in a clinical setting, have reduced temporal variability in LVEF measurements by improving intra- and interobserver variability and test-retest variability, which is important in CRTCD where serial evaluation of LV systolic function is needed. Despite these advances, the utility of LVEF in CTRCD is still limited since changes of <10 percentage points between examinations do not necessarily represent an actual change in systolic function (11). Furthermore, changes in LVEF are a late manifestation of CTRCD, hence LVEF has a low sensitivity for detecting early subclinical changes in LV function.

**Figure 1:**
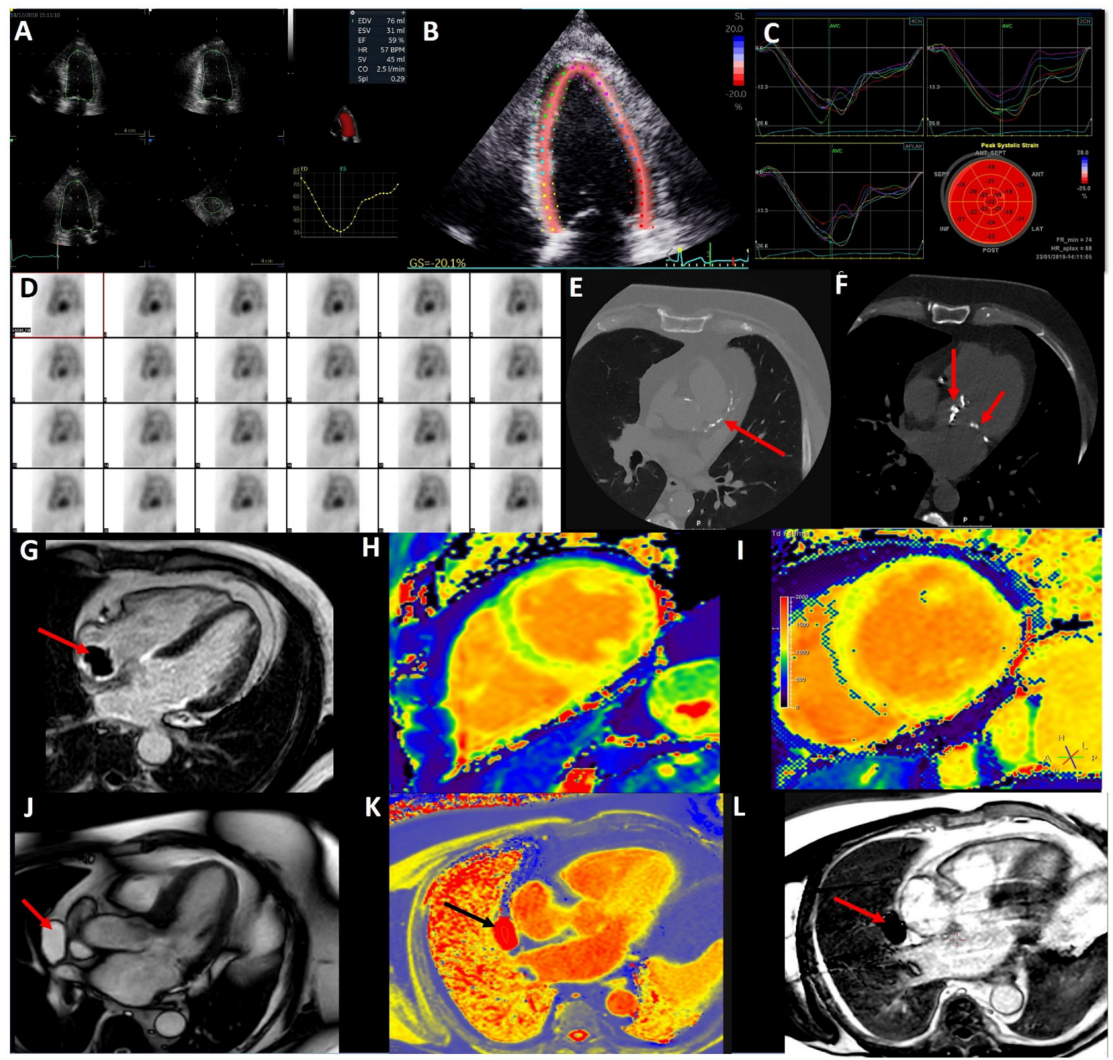
Multi-modal imaging in cardio-oncology. **(A)** 3D echocardiography to accurately calculate left ventricular volumes and ejection fraction. **(B)** 2D Speckle Tracking Echocardiography of the left ventricle (4 chamber view) for Global Longitudinal Strain. **(C)** 2D Left Ventricular Global Longitudinal Strain curves from 4, 2, and 3 chamber views. **(D)** Nuclear medicine—Multi-gated blood pool imaging to determine LVEF. **(E)** CT coronary angiogram demonstrating coronary artery calcium in the left anterior descending artery in a lymphoma survivor. **(F)** CT coronary angiogram demonstrating radiotherapy related aortic and mitral valve calcification in the same patient. **(G)** Cardiac Magnetic Resonance (CMR) late gadolinium enhancement with long T1 inversion time demonstrating a thrombus (red arrow) on the end of a Hickman's line in a cancer patient. **(H)** Normal CMR T1 map (green is normal myocardium). **(I)** T1 map showed elevated T1 times of the left ventricle in myocarditis. **(J)** CMR 3Ch cine demonstrating a pericardial mass (red arrow). **(K)** CMR T1 map highlight the pericardial mass is fill with fluid (black arrow). **(L)** CMR LGE with long T1 inversion time demonstrating mass (red arrow) is avascular with no enhancement.

Speckle tracking echocardiography, which has been validated against sonomicrometry and tagged magnetic resonance imaging, has provided accurate angle-independent measurements of myocardial strain. Efforts to standardize myocardial deformation imaging has reduced the variability in this measure compared to other conventional echocardiographic measures of LV systolic function making it ideal for CTRCD (12). Global longitudinal strain (GLS) has been shown to detect LV dysfunction earlier than LVEF in patients receiving cancer therapy and has the potential to guide therapy (13). GLS has better inter and intra observer reproducibility than 2D LVEF by biplane method of disks emphasizing recent society statements that encourage the use of GLS and 3D LVEF in baseline echocardiographic assessments of cancer patients (see Figures 1A–C) (7, 14). GLS and 3D LVEF have the lowest temporal variability with respect to the detection of CRTCD. In a group of hematological cancer patients undergoing anthracycline therapy with normal LVEF, those with a baseline GLS <-17.5%, were associated with a six-times higher increase in cardiac death or symptomatic heart failure (15).

The latest guidelines define CTRCD as a LVEF drop of ≥10% to a value below the lower limit of normal (<50%) (5, 16). Risk stratification is key to determining surveillance strategy, to ensure patients undergo optimal cancer therapy whilst minimizing the risk of CTRCD. High risk treatment factors are simultaneous doxorubicin and trastuzumab, high-dose doxorubicin (≥400 mg/m^2^ or equivalent), ≥30 Gy of radiotherapy to the chest involving the heart and tyrosine kinase inhibitors following doxorubicin chemotherapy (9). High risk patients are those with underlying cardiovascular disease, numerous cardiac risk factors, age ≥65 years, impaired LV function and previous cardiotoxic therapy (9). Those that are high risk should have imaging surveillance every two cycles or every cycle above 240 mg/m^2^ of doxorubicin or equivalent (9). In regards to frequency of serial echocardiograms, it is dependent on the type and dose of anticancer agent as well as symptoms (5).

**Figure 2:**
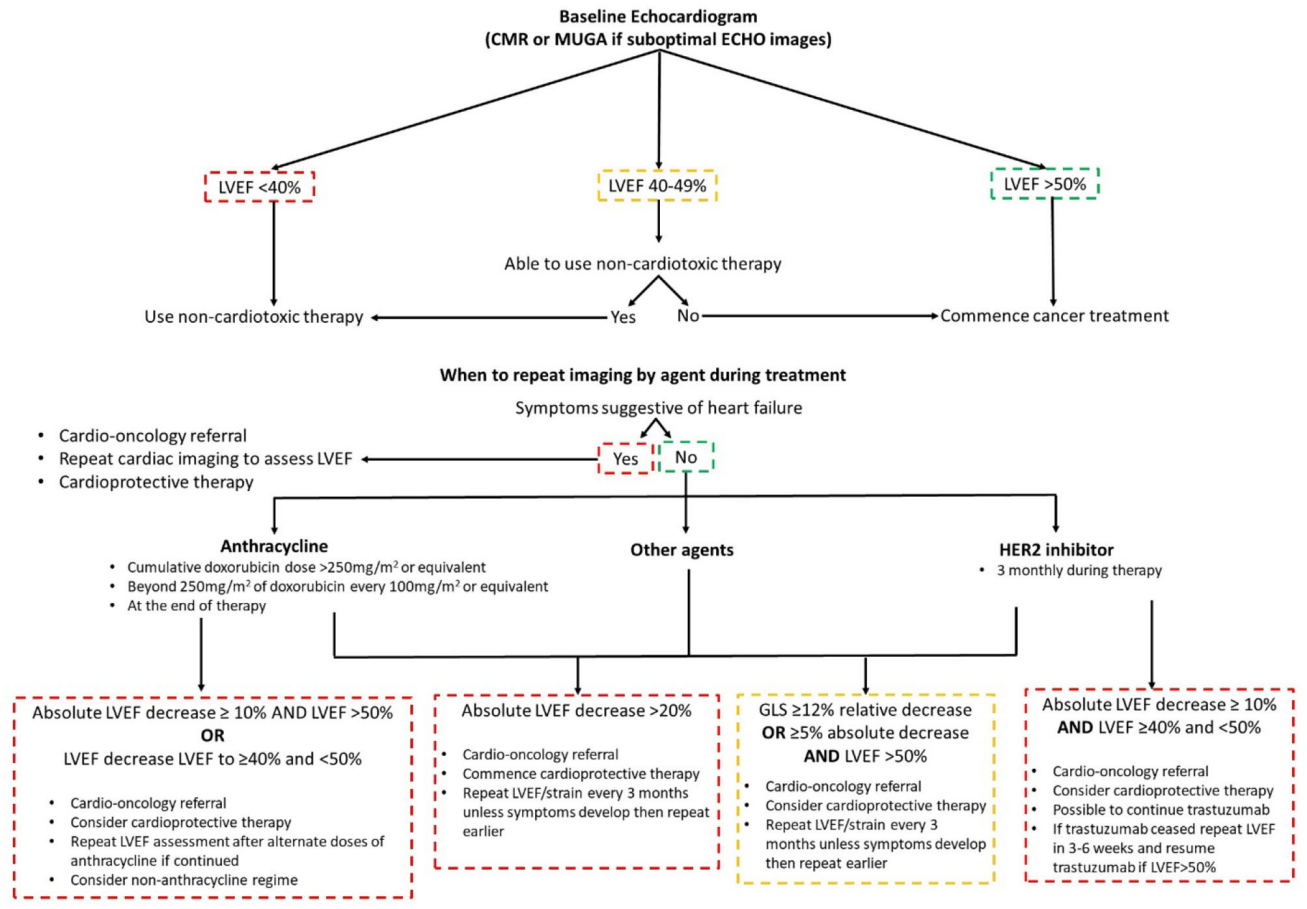
Summary of the ESMO 2020 cardiac imaging guidelines.

The value of imaging surveillance and risk stratification was highlighted in patients treated with anthracyclines and HER2 inhibitors, with a decline in GLS reported as early as 3 months after the initiation of trastuzumab in the adjuvant setting (13, 17–19). GLS has a good prognostic ability for detecting CTRCD and the latest guidance stipulate a 12% relative decrease or a ≥5% absolute decrease in GLS with normal LVEF should trigger the treating physician to consider cardioprotective therapy and a repeat LVEF and strain measurement in 3 months if asymptomatic (Figure 2) (5). Cardioprotective therapy consists of angiotensin converting enzyme inhibitors (ACEi), beta blockers (BB) and dexrazoxane (5). Recently the Strain Surveillance of Chemotherapy for Improving Cardiovascular Outcomes (SUCCOR) study published its 1-year data (20). Though the SUCCOR primary outcome of change in LVEF was not significantly different between a GLS and 2D LVEF treatment strategy (*p* = 0.05), patients in the GLS arm had higher cardioprotective therapy rates and fewer developed CTRCD (6 vs. 14%; *p* = 0.02) (20).

CTRCD recovery is dependent on early detection. Those who start treatment with a cardioprotective agent(s) within 2 months of diagnosis have a better chance of LV function recovery (21). Additionally, those that start CTRCD treatment with a lower LVEF may have a lower chance of recovery in LVEF (22). In an anthracycline and trastuzumab breast cancer cohort, patients who had reversible CTRCD had higher nadir GLS compared to those with irreversible CTRCD (−17 vs. −11.7%, respectively) (23). This emphasizes the importance of cardiac imaging and the need for improved and better imaging parameters in CTRCD. A future potential echocardiographic parament is 3D LV strain. It includes no through-plane motion of speckles and the ability to track speckles in 3D space. This permits the calculation of circumferential, radial, and longitudinal strain in one measurement. Though an exciting prospect, more studies are needed in this area.

#### Left Ventricular Diastolic Function

Baseline LV diastolic function is not predictive of CTRCD and there is limited evidence on it predicting CTRCD (24). The largest prospective study to date demonstrated a worsening in diastolic function from baseline is associated with a 1.4% decrease in LVEF from baseline with a 2.2 times increased risk of developing CTRCD (24).

#### Right Ventricle

Though still limited by small studies, quantitative assessment of the RV size and function is recommended as RV dysfunction can occur during cancer treatment (7). In addition to standard parameters, 2D RV strain is recommended in the latest joint society guidelines (25). The data on RV strain in CTRCD suggests RV dysfunction frequently occurs with LV dysfunction and pertains a reduced recovery in LV function, though further studies are needed (26). Additionally, other studies suggest 2D and 3D RV strain deterioration occurs prior to LV parameters (27, 28).

#### Pumonary Hypertension

Tyrosine Kinase Inhibitors (TKI) (e.g., dasatinib) are associated with pulmonary hypertension, though the incidence is difficult to estimate because of lack of screening data due to asymptomatic study participants and overall small study sizes. A position statement suggests 3–6 monthly ECHOs in asymptomatic patients on anticancer agents that can cause pulmonary hypertension such as TKIs (16). Dasatinib induced pulmonary hypertension is often reversible, but not to normal baseline pulmonary pressures highlighting the importance of identifying a better method to find at risk patients earlier (29).

#### Stress Echocardiography

The role of stress echocardiography is potentially useful in risk stratifying patients undergoing cancer therapies associated with ischemia. This includes antimetabolites (5-FU), VEGF inhibitors and TKIs (30). The advantage of exercise or pharmacological stress echocardiography is no radiation, high feasibility, and low cost. The role of stress echocardiography beyond ischemia testing to identify CTRCD using parameters such as diastolic dysfunction is inconclusive (31, 32).

#### Left Atrium

Aspects of the left atrium that have been investigated include baseline left atrial volume index and left atrial longitudinal strain (33, 34). Bergamini et al. found that a dilated left atrium with normal LV function on baseline echocardiography prior to adjuvant or neoadjuvant traztuzumab is associated with the development of CTRCD in patient receiving trastuzumab (33). Park et al. found peak atrial longitudinal strain decline at the end of chemotherapy could predict CTRCD with better sensitivity and specificity than LV GLS (34).

### Long-Term Cardiac Complications in Cancer Survivors

Long-term cardiac surveillance is recommended post completion of chemotherapy (9). In low to medium risk patients, a 12 months post final treatment echocardiogram is recommended with 5-yearly reviews if asymptomatic (9). In high risk groups 6 and 12 months post final cycle review followed by annually for 2–3 years is initially recommended (9). In patients with symptomatic CTRCD annual review with echocardiography is recommended (7). Female survivors of childbearing age who have had cardiotoxic therapy/chest radiotherapy should have a cardiology consultation prior to pregnancy with echocardiographic surveillance performed in the first trimester (35). It is important to note, 10–20 years post anthracyclines therapy nearly 50% of patients will show evidence of CTRCD and experience reduced quality of life and a mortality similar to dilated cardiomyopathy patients (36–38). Reassuringly, modern anthracycline regimes appear to have a lower impact on LVEF (39).

A collection of echocardiographic parameters rather than solely LVEF maybe important in identifying at risk patients, as the prevalence of abnormal GLS and diastolic function is higher in a cancer survivor cohort (40). This is supported by another long-term childhood cancer survivor cohort, which demonstrated significantly impaired RV function (41).

## Cardiac Magnetic Resonance

### Baseline Cardiovascular Risk Stratification

CMR is the gold standard for measuring left and right ventricular volume and function (42, 43). However, its major limitation is it is high cost and lack of availability relative to echocardiography (44).

The main indication in the current cardio-oncology guidelines for CMR is when there is suboptimal image acquisition and it is preferred over nuclear imaging (6, 7, 16). A key strength of CMR is in the assessment of cardiac masses and inflammatory conditions such as myocarditis, pericarditis, and myopericarditis. This is due to its advantages of multi-planar image acquisition, high spatial resolution, a large field of view, and tissue characterization (45). Tumors and thrombi can be easily differentiated with difference CMR sequence. Tumors tend to be hyperintensity on T2-weighed turbo spin echo, contrast first pass perfusion, and late gadolinium enhancement (LGE), whereas thrombi are hyperintensity with short T1 and hypointensity with long T1 times (see Figure 1G) (46).

Using the forementioned CMR sequences, CMR has a high accuracy for discriminating between benign and malignant lesions, with a good interobserver agreement (47). Figures 1J–L show a pericardial cyst, identified by different CMR techniques. The differentials for the cyst are a pericardial diverticulum and mediastinal mass.

The role of CMRs in cardio-oncology from current patients to survivors is likely to increase given its high reproducibility and lack of ionizing radiation. This may be enhanced with the potential development of a limited 10 min CMR examination focusing on volumes and function (48). This would invariable lower the cost and increase the availability of CMR.

### Identification of CTRCD and Predicting Recovery

Though CMR main role in baseline assessment is limited, its versatility makes it an important imaging modality in the identification of CTRCD.

#### Cardiac Dysfunction

The definition of CTRCD is based on a LVEF decline of >10%, reiterating the importance of accurate and reproducible imaging. CMR is superior to 2D echocardiography in identifying LV dysfunction as its volumes are not based on geometric assumptions and less prone to suboptimal imaging (49). Furthermore, it has superior reproducibility compared to echocardiography (43).

CMR can also measure myocardial strain. The data on CMR myocardial strain correlates with a decline in LVEF, however its prognostic ability has not been assessed to the extent it has been in echocardiography (50–53).

#### Late Gadolinium Enhancement

The composition of the extracellular matrix is altered in myocardial fibrosis. This structural change allow gadolinium to accumulate in areas of replacement fibrosis. On T1-weighted sequences regions of gadolinium accumulation appear hyperintense (bright) in contrast to healthy myocardium, which appear dark. The data on the utility of LGE in CRTCD is conflicting. The majority of short term (<6 months) studies have not reported any LGE (51, 53, 54). Longer term follow up studies document LGE incidence of 5–19% (55, 56). The largest and most recent study to date on LGE and anthracyclines +/- trastuzumab identified a LGE incidence of 10% with an alternative cause for LGE identified in nearly all cases, calling into question the value of LGE in identifying CRTCD secondary to anthracyclines and/or trastuzumab (57).

#### Tissue Characterization

The utility of tissue characterization has becoming increasingly popular over the last decade with the advent of validated software enabling the quantification of T1, T2 mapping, and extracellular volume (ECV) fraction estimation. T1 mapping allows us to detect a range of diffuse pathologies including myocardial fibrosis, myocarditis, cardiac amyloidosis, key aspects of cardio-oncology as well as other pathologies such as storage disorders. T1 measures the longitudinal time to equilibrium post a radiofrequency pulse. The commonest technique to acquire a T1 map is the modified Look-Locker pulse sequence (MOLLI) or “shortened” version known as the shMOLLI (58). Though derived T1 values, the main advantage of these techniques are reduced acquisition time and the shMOLLI in particularly has shorter breath-holds. Native T1 mapping refers to the acquisition of a T1 map without contrast (Figures 1H,I). It is important to note T1 values vary from scanner to scanner and tesla strength (59). The literature on T1 mapping identifying CTRCD are conflicting and limited with the focus being on survivors. The three human studies to date show differing changes in T1 value shortly post anthracycline exposure, with two showing increases in T1 values whilst the other identified a decrease in T1 at 48 h post anthracycline as predictive of CTRCD (54, 60, 61).

ECV is an additive tool in the assessment of myocardial fibrosis assessing interstitial fibrosis. ECV requires a pre- and post-contrast T1 map as well as the subject's hematocrit to calculate the ECV fraction. Normal values are between 20 and 26%, with it being slightly higher in women and similar between 1.5T and 3T scanners (59, 62, 63). There are only two human studies to date assessing ECV changes during chemotherapy with one showing a significant temporal change in those developing CTRCD whilst the other showed no significant changes in ECV fraction (54, 61). Furthermore, the temporal variability of T1 mapping and ECV was comparable to those with no CTRCD (61).

T2 measures the transverse time to equilibrium post a radiofrequency pulse. T2 mapping is used in the identification of myocardial oedema, which can occur in myocarditis to infarction to cardiomyopathy. Its use along with T1 mapping is supported in myocardial inflammation recommendations (64). The data on T2 is limited to two human studies with conflicting results (54, 61).

The data on T1 time and ECV in cancer survivors are heterogenous. Increases in T1 and ECV correlated to cumulative dose, reduced exercise capacity and myocardial wall thinning in cancer survivors whom received anthracycline and were at least 7 years in remission (65). Additionally, the ECV fraction is further increased in those with reduced LVEF (66). However, other studies have found no significant increase in native T1 and ECV in childhood cancer survivors (67, 68). The role of tissue characterization in CTRCD requires further studies given the limited and conflicting results to date.

##### Myocarditis

Though immune checkpoint inhibitor (ICI) related myocarditis is rare, its frequency will increase with increased ICI usage. CMR is a valuable non-invasive diagnostic tool in the assessment of myocarditis with the Lake Louise criteria and the addition of T1 and T2 maps (Figure 1I) (64). Its role has been supported in the workup of ICI myocarditis (69). The largest study to date, a registry of 136 ICI myocarditis patients showed abnormal T1 values were associated with more symptoms, lower cardiac function (70). Furthermore, higher T1 values had independent prognostic value for the subsequent development of major adverse cardiac events (70). Elevated T1 times were commoner than elevated T2 times at 78 and 43% of patients, respectively, however all patients met the modified Lake Louise criteria (70).

## Computer Tomography

### Baseline Cardiovascular Risk Stratification

The calcium artery calcium (CAC) score can be determined on non-gated non-contrast CT chest scans performed for staging and assessment of the primary malignancy, this CAC should be reported as per guidelines (71). The concept of higher CAC score correlates with higher acute coronary events has been reaffirmed in the lung cancer screening and breast cancer populations (72, 73). A recent study using an automated CAC algorithm in >14,000 breast cancer patients has demonstrated a CAC score >400 is associated with a five times higher risk of cardiovascular death compared to a zero CAC (74). Though the role of CT is limited in cardio-oncology guidelines, CT coronary angiography may serve as an alternative imaging modality to stress echocardiography in a baseline assessment, though may be limited by its higher cost and radiation exposure.

### Long-Term Cardiac Complications in Cancer Survivors

#### Coronary Artery Disease

Complications of radiotherapy include accelerated atherosclerosis (75). Effects are often identified in the medium to long term. There is a linear radiation dose to risk of CAD relationship, with the excess relative risk of CAD per mean heart Gray dose being 7% (76). Whether radiotherapy leads to an increase visible CAC on CT is contested (77, 78). Figure 1E illustrates extensive CAC in a lymphoma survivor. In regard to acute coronary syndrome, the main risk factor is the volume of LV receiving 5Gy and for each Gy the cumulative incidence increase of acute coronary syndrome is 17% (78). Despite advances in radiotherapy technology and techniques to minimize cardiac complications, close surveillance of radiotherapy therapy patients is warranted 5–10 years post radiotherapy (79).

#### Valvular Disease

Radiotherapy can impact the valvular apparatus causing thickening, fibrosis, and significant valvular heart disease (75). The risk of valvular disease from radiotherapy is 34 times higher and occurs in the second decade post treatment (80). The role of CT has been elevated with ability to assess of aortic valve calcium scoring (81). Figure 1F illustrates an example of aortic and mitral valve calcification post radiotherapy in a lymphoma survivor. Radiotherapy patients experience mediastinal damage, such as mediastinal fibrosis and porcelain aorta, which can make cardiac surgery more complicated.

#### Pericardial Disease

Acute and chronic pericarditis are side effects often associated with mediastinal radiotherapy, though can also occur with chemotherapy (75). Their incidence is related to the cumulative radiation dose. The incidence of radiation induced pericarditis has significantly decreased to 2.5% due to advances in radiotherapy techniques and shielding methods (82). In chronic pericarditis calcification of the pericardium may occur. CT is the ideal modality for assessing pericardial calcification and thickening due to its excellent spatial resolution (83).

## Nuclear Imaging

Multi-gated blood pool imaging (MUGA) was the first imaging modality to be used in cardio-oncology (84). Its primary role historically has been in the measurement of the LVEF (see Figure 1D). MUGA's LVEF measurement was reported superior to 2D echocardiography in 1980's, however, is inferior to CMR (85, 86). Its limitation is the relatively high radiation exposure (5–10 mSV) and the inability to assess other cardiac parameters, has led to a decline in its usage (7). However, there is a role for ^18^F fluorodeoxyglucose (^18^FDG) positron emission tomography (PET)-CT scans particularly in the diagnosis and staging of cancer patients. Inflammation is associated with both cancer and cardiovascular disease. ^18^FDG-PET can identify cardiac inflammation within atherosclerotic plaque as well as myocardial tissue and valves (87, 88). Retrospective data in patients who have received doxorubicin and a subsequent increase in LV ^18^FDG uptake, are associated with a decline in LVEF (89). This increased uptake suggests there may be a myocardial inflammation component to CTRCD and warrants further investigation despite its high cost and lower availability. With the increasing use of ICIs myocardial inflammation will become an increasing problem. The limited evidence to date indicates ^18^FDG-PET does not identify ICI related atherosclerosis, however further investigation is warranted, potentially with more specific tracers for vascular inflammation (87, 90).

## Future Directions

All cardiac imaging modalities have a promising role in the future of cardio-oncology. Areas of particular interest that may rise to prominence, are advanced echocardiographic assessment of cardiac structures in addition to the left ventricle in conjunction with the use of 3D volumes and myocardial deformation indices over standard 2D echocardiography as described earlier. Similarly, the utility of diffusion tensor CMR can increase due to its ability to assess myocardial microstructure, such as, cardiomyocyte and sheetlet-level *in vivo* (91). As CT technology and techniques evolve, CT may potentially play a more significant role in tissue characterization such as ECV as well as chamber volume assessments should radiation doses reduce sufficiently (92, 93). Despite nuclear imaging's higher radiation dose, it will continue to play an important role in certain subsets of cancer patients where it can be utilized to monitor patients' baseline cancer stage and progression. Incorporating more cardiac specific parameters such as LV ^18^FDG uptake may identify new CTRCD markers at no inconvenience to patients. Lastly, contrast-enhanced ultrasound (CEU) molecular imaging could become a novel imaging modality in cardio-oncology. CEU can track temporal and spatial changes in tissues and the vasculature, which is important, as anthracyclines can damage the myocardial vascular bed by labeling molecular markers with specific antibodies (94, 95). Though this is currently limited to research, the transition to clinical use warrants monitoring.

## Conclusion

The field of cardio-oncology has evolved rapidly over the last couple of decades. Cardiac imaging has an integral role in this specialty and advances in imaging techniques allow clinicians to identify at risk patients earlier. Each imaging modality has its pros and cons with no sole technique superior in all domains of cardiac imaging. Thus, the key to a successful cardio-oncology patient journey involves a multi-modal approach so that patients can hopefully complete their optimal cancer therapy with minimal disruption to their cardiac health.

## Author Contributions

All authors have contributed to the planning, writing, and reviewing of the manuscript.

## Conflict of Interest

The authors declare that the research was conducted in the absence of any commercial or financial relationships that could be construed as a potential conflict of interest.

## Publisher's Note

All claims expressed in this article are solely those of the authors and do not necessarily represent those of their affiliated organizations, or those of the publisher, the editors and the reviewers. Any product that may be evaluated in this article, or claim that may be made by its manufacturer, is not guaranteed or endorsed by the publisher.
