# Supplementary material for: The Utility of Advanced Cardiovascular Imaging in Cancer Patients—When, Why, How, and the Latest Developments
Source: Front Cardiovasc Med. 2021 Sep 3;8:728215. doi: 10.3389/fcvm.2021.728215 (PMC8446374; doi:10.3389/fcvm.2021.728215)
Supplement: Supplementary file 1 [file Table_1.DOCX]

| **Supplementary Table 1: Summary of cardiotoxic agents, their effect and cardiac imaging modality for each cardiotoxicity** | | | | | |
| --- | --- | --- | --- | --- | --- |
| **Cardiotoxic agent** | **Cardiotoxic effect** | | | | |
|  | **Cardiac dysfunction** | **Myocardium** | **Pericardium** | **Valvular disease** | **Coronary artery disease** |
| **Conventional agents** |  |  |  |  |  |
| Anthracyclines (doxorubicin) | ✔ - ECHO, CMR, MUGA | - | - | - | - |
| Antimetabolites (5-FU) | ✔ - ECHO, CMR, MUGA | - | - | - | - |
| Alkylating (cyclophosphamide) | ✔ - ECHO, CMR, MUGA | - | - | - | - |
|  |  |  |  |  |  |
| **Targeted agents** |  |  |  |  |  |
| HER2 inhibitors (trastuzumab) | ✔ - ECHO, CMR, MUGA | - | - | - | - |
| Tyrosine Kinase Inhibitors |  |  |  |  |  |
| VEGF (sunitinib | ✔ - ECHO, CMR, MUGA | - | - | - | - |
| EFGR (osimertinib) | ✔ - ECHO, CMR, MUGA | - | - | - | - |
| ALK (crizotinib) | ✔ - ECHO, CMR, MUGA | - | - | - | - |
| Proteasome inhibitors  (bortezomib) | ✔ - ECHO, CMR, MUGA | - | - | - | - |
|  |  |  |  |  |  |
| **Immunotherapy*** |  |  |  |  |  |
| Immune checkpoint inhibitors  (ipilimumab) | ✔ - ECHO, CMR, MUGA | ✔ - CMR | ✔ - ECHO, CMR, CT | - | - |
| **Radiotherapy** | - | ✔ - CMR | ✔ - ECHO, CMR, CT | ✔ - ECHO | ✔ - CTCA |

ECHO – echocardiogram, CMR – cardiac magnetic resonance, MUGA – Multi-gated blood pool imaging CT – Computer tomography, CTCA – Computer tomography coronary angiogram. *Troponin and electrocardiogram are important non imaging tests in diagnosing cardiotoxicity in patients who use these agents.
